# Energy Efficient Node Selection in Edge-Fog-Cloud Layered IoT Architecture

**DOI:** 10.3390/s23136039

**Published:** 2023-06-29

**Authors:** Rolden Fereira, Chathurika Ranaweera, Kevin Lee, Jean-Guy Schneider

**Affiliations:** 1 School of Information Technology, Deakin University, Geelong, VIC 3220, Australia; rjfereira@deakin.edu.au (R.F.); kevin.lee@deakin.edu.au (K.L.); 2 Faculty of Information Technology, Monash University, Clayton, VIC 3168, Australia; jean-guy.schneider@monash.edu

**Keywords:** IoT, energy, edge computing, cloud, fog, node selection, optimal, ILP

## Abstract

Internet of Things (IoT) architectures generally focus on providing consistent performance and reliable communications. The convergence of IoT, edge, fog, and cloud aims to improve the quality of service of applications, which does not typically emphasize energy efficiency. Considering energy in IoT architectures would reduce the energy impact from billions of IoT devices. The research presented in this paper proposes an optimization framework that considers energy consumption of nodes when selecting a node for processing an IoT request in edge-fog-cloud layered architecture. The IoT use cases considered in this paper include smart grid, autonomous vehicles, and eHealth. The proposed framework is evaluated using CPLEX simulations. The results provide insights into mechanisms that can be used to select nodes energy-efficiently whilst meeting the application requirements and other network constraints in multi-layered IoT architectures.

## 1. Introduction

With diverse IoT applications being introduced every day and a prediction of a significant increase in the number of IoT devices, there is a demand for a stable and scalable IoT infrastructure to accommodate futuristic IOT use cases. Current technological innovations in computation and communication technologies are pivotal in defining an IoT communication infrastructure for supporting this exponential growth. A combination of 5G/6G wireless communication, edge, fog, cloud computing, software-defined networking, and artificial intelligence would help support this growth in the IoT network [1]. On the other hand, the increasing energy demand, exponential increase in energy cost in IoT, and its environmental impact have diverted industries towards identifying the best feasible ways to control, manage, monitor, and save energy in IoT architectures. However, providing a cost-effective and energy-efficient scalable infrastructure for emerging IoT applications by incorporating these vast heterogeneous communications and other emerging technologies has become a significant challenge. It is mainly because each technology has its requirements, architectures, and frameworks. It is important to be cautious when integrating these advanced technologies, to effectively support emerging IOT use cases in a way that saves energy and keeps the cost reasonable [2].

In the past few years, the research community has introduced use case-centric IoT architectures and emphasized meeting quality of service (QoS) constraints for a single IOT use case and its sub-applications. These architectures typically comprise edge or cloud technology and a communication network [3]. The QoS and network parameters considered, include time synchronization, service accuracy, service priority, availability, response time, reliability, delay, throughput, and security [4,5]. Apart from these parameters, researchers have also highlighted the importance of task offloading and energy efficiency in an IoT architecture in achieving sustainable IoT deployment and operations [6,7].

To gain the full benefits of emerging IoT applications and to achieve cost-effectiveness and energy efficiency, upcoming computation, communication, and caching mechanisms need to be converged intelligently, considering all general IoT applications rather than assuming a single use case [8]. A generalized flexible IoT network architecture is required, which would be feasible to serve all the IoT use cases and their sub-applications that can meet the QoS and application constraints. However, achieving energy-efficient operations combining diverse communication and computation technologies while satisfying emerging user application QoS and network requirements, has received minimal attention.

In this paper, we have considered a distributed IoT architecture comprising edge, fog, and cloud layer connected to heterogeneous appliances/gadgets at the edge layer, serving diverse IoT use cases. Each layer consists of different nodes, and each node is equipped with a custom number of servers that can perform various IoT requests. We also propose an Integer Linear Programming (ILP)-based optimal node selection framework that can minimize the energy consumption of the IoT network when selecting a node for processing a new IoT request while meeting the IoT application and network requirements. The framework considers the energy consumption of processing an IoT application at all three layers, edge-fog-cloud. The framework is evaluated using CPLEX simulations considering diverse IoT requests from use cases encompassing eHealth to autonomous vehicles.

The pivotal benefactions of this paper can be summarized as: (1) The exploration of efficient IoT architecture comprising of edge, fog, and cloud layer for computation; (2) the suggestion of optimal node selection technique to minimize the energy in the IoT network architecture while fulfilling the constraints of IoT application and the limitations of the connectivity network; (3) consideration of a custom number of servers deployed at each node when selecting a node for processing new requests instigating from diverse IoT applications with varying requirements; (4) providing insight into how the energy cost affects the optimal selection of nodes.

The rest of this paper is ordered as follows. Section 2 presents a literature review on the IoT architectures, task offloading, node selection mechanisms and frameworks for emerging, advanced IoT applications and their energy management. In Section 3, we elaborate on the research challenges in the node selection in heterogeneous IoT architecture whilst achieving the energy efficiency. In Section 4, we explore a heterogeneous IoT network architecture and present a comprehensive description of a suggested optimal node selection framework. Our formulation includes mathematical details and aims to achieve energy-efficient operation within the IoT network. Section 5 of the paper presents an assessment of the proposed framework and Section 6 presents the comments on the entire paper and the proposed framework while the concluding remarks can be found in Section 7.

## 2. Background on IoT Architectures and Energy Management

This section provides a concise overview of past research on IoT architectures and associated frameworks, which have been designed to facilitate diverse use cases and enhance energy efficiency.

### 2.1. IoT Architectures

**Figure 1:**
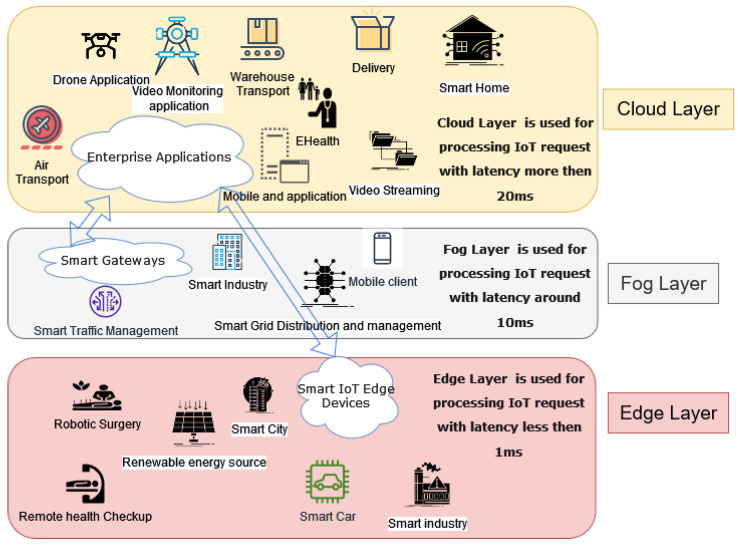
Edge-Fog-Cloud layers in IoT.

IoT architectures have mainly used three computation layers, edge-fog-cloud for on-demand services, and to provide shared and distributed resources to diverse IoT applications. IoT applications are used in many sectors, including defense, healthcare, smart cities, industrial automation, and farming. Each of these applications use a single layer for computation depending on the application requirements [9]. Figure 1 shows diverse applications used in each layer. By utilizing pooled, virtualized, and scalable resources, as well as flexible services and scalable storage, the cloud layer facilitates distributed computing, which is bolstered by managed and controlled computing power [2]. The fog layer, which offers virtualized, flexible, and adaptable operation of computation resources, network management, and repository services, is a modern layer that is located closer to the user than the cloud. It is gaining traction as an emerging technology and is expected to play a crucial role in the future of computing [10]. It mainly serves to reduce latency and conserve bandwidth, with network security enhancement for IoT applications. On the other hand, edge computing is an emerging computational technology where the computation is carried out in the vicinity of the data sources [2,10]. Edge computing provides computational potential at the edge, to the increasing IoT devices and provides a solution to the limitation of cloud computation in processing the data closer to the user, enabling low latency communication.

### 2.2. Task Offloading and Node Selection in IoT networks

With the availability of a limited amount of computation and communication resources in an IoT architecture, it is necessary to implement methods for achieving optimal utilization of resources and performance optimization. With an exponential increase in the IoT requests from billions of IoT devices, we would require task offload and sharing mechanisms to manage the available resources. Therefore, efficient task offloading, load balancing, and resource-sharing mechanisms have been investigated to offload the tasks and select the most efficient resources for processing, while achieving the required QoS, reliability, and energy efficiency.

#### 2.2.1. Task Offloading

Task offloading mechanisms in IoT architectures emphasize sharing the load of the IoT requests coming in from various IoT use cases, among the resources available across multiple computation layers. It ensures that there is efficient utilization of local resources and the resources available nearby, with the minimum energy usage for transmission [11]. In [12,13], researchers have suggested switching off/on device techniques in fog nodes for task offloading, which would help save energy. The primary focus of an efficient task offloading mechanism would be on balancing the tasks among available computational resources. Hence, we need to consider load balancing for data management in the edge-fog-cloud layer, to provide the anticipated quality of services. Load balancing algorithms are also termed as task scheduling and task offloading algorithms. In load balancing algorithms, the cluster technique and bee colony have been used to decrease the load of the system with the usage of virtual machines [14]. Several traditional optimization techniques, including ACO, for load balancing, are also proposed to balance the load in smart grid cloud computing systems [14]. Further, ACO and particle swarm optimization (PSO) are also used to effectively load balance IoT tasks at the fog nodes under constraints including communication cost and response time [14]. Edge computing deployment can be achieved using a mobile resource-sharing framework that relies on mobile edge servers wherein the edge resources could be shared by multiple IoT devices [15]. These frameworks that focus on task offloading and load balancing emphasize reducing the resource utilization and load at a single layer and lack consideration of all the computation layers.

#### 2.2.2. Node Selection

The location of the nodes is critical for both resource allocation and ensuring that end users’ QoS requirements are met. Different proposals reported mechanisms for fog node placements. For example, the fog node placement framework has been proposed in [16] in which traffic aggregation is used to optimize the number of fog nodes deployed and minimize latency. The author also identified that it would not be ideal to store all the locations of fog nodes and iterate the list to select each fog node every time a request comes in. Another critical factor we need to consider is the active node selection, which is vital in resolving issues like resource allocation, network lifetime, and data integrity.

The optimal fog node selection can also meet low latency demands and identify anomalies in IoT networks. For example, authors in [17] proposed an unsupervised machine learning-based mechanism incorporating k-means clustering with Principal Component Analysis (PCA) for selecting nodes for meeting latency requirements. In [18], resource utilization in the fog-edge layer was achieved using a profitable resource allocation scheme. The number of nodes to process a request was decided using optimal node placement at the fog layer. Batch processing of application placement in concurrent IoT applications in fog/edge environments using the memetic algorithm was proposed in [19]. The authors showed that the proposal minimized the execution time and energy consumption of IoT applications. To place the most popular service as close to the user as possible using the fog layer under the constraint of minimizing latency was proposed in [20,21]. Hence to achieve this, user mobility pattern prediction and migration using virtual machines was used for optimized request placement in the fog. In fog IoT architecture, path selection optimization was achieved by breaking down application based on platform as a service (PaaS) into components among fog and cloud layer in [7]. By using control parameters like deadline, threshold capacity, cost and latency, virtual network functions (VNF) was implemented via application graphs. In [22], the Q-learning-based reinforcement learning method with ascendant gradient was used for node selection in a fog-cloud IoT architecture. The allocation was accomplished without using any preceding information about the system and allocating the widely distributed resources under control parameters like average request arrival rate, transfer rate, job size, process proportion spread. Most of the research on fog-based IoT architectures has focused on optimizing a specific QoS metric, and the optimization technique used for enhancing the performance of the IoT architecture is very specific to an IoT application use cases. As categorized in the above section, most researchers have focused on task offloading and node selection because of the limitations on the availability of resources and an exponential increase in data being generated and requests coming through. The researchers have emphasized various IoT architectures at different layers to meet the resource requirements. The research in resource allocation and node selection has highlighted energy efficiency, bandwidth, and task scheduling as objective functions and has used the Markov determination procedure, poly-time algorithm, Markov decision process, Q-learning based reinforcement algorithm with control parameters like threshold, capacity, cost, latency, data rate, average request arrival rate, transfer rate, job size, and CPU capacity. For fog-based node selection and application placement, the researchers have emphasized path selection, collating fog nodes and optimized service placement as objective function using methodologies like linear programming, ifogsim, icloudsim, cloudify, and control parameters like deadline threshold, cost, data rate, latency, network usage, and energy consumption.

In the Edge Computing (EC)-based IoT architectures, it is observed that a system with more EC performs better compared to a system with less EC [23], but the usage of static resources increases the cost of running IoT services too. It is also identified that another significant challenge in fog computing and edge computing-based IoT architectures is the optimal allocation of nodes for workload management to meet the Service Level Agreement(SLA) and QoS parameters. An optimal node selection framework has been introduced by authors based on the usage of all three layers cloud, fog and edge in an IoT architecture [24].

### 2.3. Energy Management in IoT Architectures

A scalable IoT architecture would be required to process the IoT request from billions of IoT devices, which would incur massive energy usage. Further, the increasing energy demand, exponential increase in energy cost, and environmental impact have diverted end-users and utility companies to focus on energy management in IoT architectures. For reducing and managing the energy consumption in the IoT architectures, researchers have proposed different techniques. For example, the authors in [25] evaluated the fog node selection methods for fog-based IoT architecture such as random selection, shortest estimated buffer, and shortest estimated latency to efficiently select the fog nodes for data transmission and thus reduce the overall energy consumption. To address the challenges of energy consumption in an IoT network based on sensor nodes, a task allocation based on clustering techniques have also been proposed [26]. The proposed framework enhances network lifetime and minimizes energy consumption by balancing task allocations.

In [27], authors proposed a joint offloading decision and resource allocation algorithm based on deep learning for addressing the problem of fog computation offloading under QoS, like delay and energy. In [28] authors proposed to use a poly-log algorithm to minimize the energy consumption in an edge layer-based IoT architecture. The most energy-efficient and least delay-restricted resource was allocated from the edge layer for efficient application placement and processing. In [4,5,28,29], energy consumption was used as one of the control parameters for application placement in fog and edge-based IoT architectures. In [4,5,28,29] authors have proposed breaking the application into components and use techniques like module mapping, poly-log, placement and scheduling algorithms to efficiently place the various application modules at optimal layers for processing.

Table 1 summarizes research undertaken in node selection using an energy-aware approach, emphasizing architectures like device-to-device, device-to-fog, fog-to-fog, fog-to-cloud, edge-cloud with the target as sensor node, fog node, IoT device. The critical problems discussed were cooperation among sensor nodes to transfer the task to fog nodes, selecting a suitable node, select a dumping device based on various parameters like energy, popularity, workload; and different performance metrics used were the amount of energy saved at the node, amount of energy consumed, delay, queue length, service time. To make the architecture more energy efficient, the researcher has focused on usage of sensor nodes, fog nodes, or user equipment. The critical problems the researchers focused on was finding the shortest path, location optimal location for nodes, improve internal communication using performance metrics like the amount of energy saved at the node, amount of energy consumed, delay, latency, and load balance.

However, for energy integration into an IoT architecture landscape, QoS metrics from communication layer as well as computation layer need to be considered for easier integration and efficient operation. Thus, the convergence of communication and computation must be taken into account using three layers, edge-fog-cloud, which would help us achieve the best of all the technologies available [31].

#### Network and Application QoS Constraints

We also need to consider the network and application QoS requirements to manage energy. Advanced IoT use cases like smart cities, intelligent transportation systems, smart health, Industry 4.0/5.0, and autonomous vehicles have a constraint on device cost, cost of deployment, network and area coverage, privacy, operating life of battery, security, and the number of supported devices. Most the IoT application can be supported via communication technologies that span from low-range wireless networks such as Wi-Fi, ZigBee, and Bluetooth to wide-area wireless networks including 4G and 5G [32,33,34,35].

The IoT use case-specific requirements vary with the application type and are summarized in Table 2. For example, the smart grid use cases need 5 Mbps–75 Mbps bandwidth and data transfer rates around 1Mbps and latency of 1 milliseconds-200 milliseconds depending on their sub-applications [1,36]. The autonomous vehicle use case requires quick processing of surrounding videos with very high data transfer rates (bandwidth between 512–1024 Gbps) and in close proximity of the end-users and ensuring the deployment of control messages with the least latency, which should be under a few milliseconds [36]. Advanced health IoT use cases consist of sub-applications such as remote health and remote robotic surgery, with data rate requirements in range of 5 Gbps–10 Gbps (bandwidth between 5 Gbps–512 Gbps). The remote health application does not require ultra-low latency in contrast to remote surgery applications, which involve remote implant monitoring and remote robotic surgery. While considering various IoT use cases, we have focused on only the IoT architecture and the requirements of the smart IoT use cases and not concerned about the type of IoT devices connected at the edge layer. We have only considered IoT devices as the devices from which the IoT requests are being generated. Though advanced communication technologies such as 5G could meet many IoT use-case requirements, the crucial challenge of supporting the exponentially increasing IoT devices and use cases still sways around. Thus, achieving complete convergence of communication and computation technologies would be necessary to defeat the communication infrastructure’s challenges.

## 3. Research Challenges and Proposal

The literature review presented in Section 2 highlights the research opportunities to provide enhanced assistance to the emerging advanced IoT use cases through full convergence of computation and communication technologies.

IoT architectures are predominantly designed with a focus on the end user, and with one specific constraint related to the IoT application. These architectures will not be adequate for encompassing all the IoT use cases with large-scale deployments for supporting the upcoming billions of IoT devices. Therefore, developing an IoT network architecture to meet the challenges of flexibility, feasibility, scalability, interoperability, and heterogeneity is crucial for synchronous and uninterrupted operation. The simulation and validation presented for the IoT architectures are limited by QoS parameters such as latency, bandwidth, resource capacity, and energy. Usage of numerical data instead of real-time data limits the evaluation process. Hence, exploring the efficiency and performance of such IoT architectures under QoS constraints such as delay, energy efficiency, reliability, service placement, and load balancing, is valuable.

The majority of the mechanisms for task offloading, energy management, and node selection described earlier focused on service placement, optimal path selection, minimizing the time for processing, reducing load by collating tasks together, or by collating the nodes to perform batch processing at either the cloud or fog layer. The earlier research has addressed edge computation using mobile edge devices as edge servers or fog nodes as edge servers. However, the optimal node selection using all three layers for computation needs further investigation in addition to the usage of stationery edge servers for processing the advanced IoT use cases. Another critical challenge is the efficient energy utilization in the IoT architectures. Energy efficiency is partially addressed in earlier research, but only in a solitary IoT use case with limited usability. Using all the three layers for computation adds extra complexities to energy usage. Hence, the efficient deployment of IoT resources focusing on energy efficiency needs further investigation.

Further, the convergence of communication and computation would be critical in providing real-time dynamic service for advanced IoT application use cases. Therefore, during the design phase of the IoT architecture, advanced communication technologies like 5G/6G, network slicing, and software-defined networks(SDN) need to be considered at the data layer, application layer, and access layer [8,37,38]. In addition, the usage of edge, fog, and cloud layers would enable collating, handling, and storage of the data at these layers dynamically in real-time.

With increasing IoT devices and an expected increase in the number of IoT requests, it would require optimal node selection for processing each request, received at the edge-fog-cloud layer. The selection of nodes for the IoT application would also be limited by the QoS metrics and network constraints of the communication architecture. An IoT network architecture with advanced communication and computation technologies can be considered to overcome the challenges. However, the next challenge in using such an architecture would be the optimal node selection mechanism to process IoT requests considering energy-efficient and cost-effective deployment of IoT resources. Hence, this paper proposes an optimal framework for node selection to process IoT requests at nodes with a varying number of server capacities and with varying network and application requirements. The framework can identify optimal nodes with the minimum energy usage for processing the IoT request at all edge-fog-cloud layers.

## 4. Optimal Node Selection Framework

**Figure 2:**
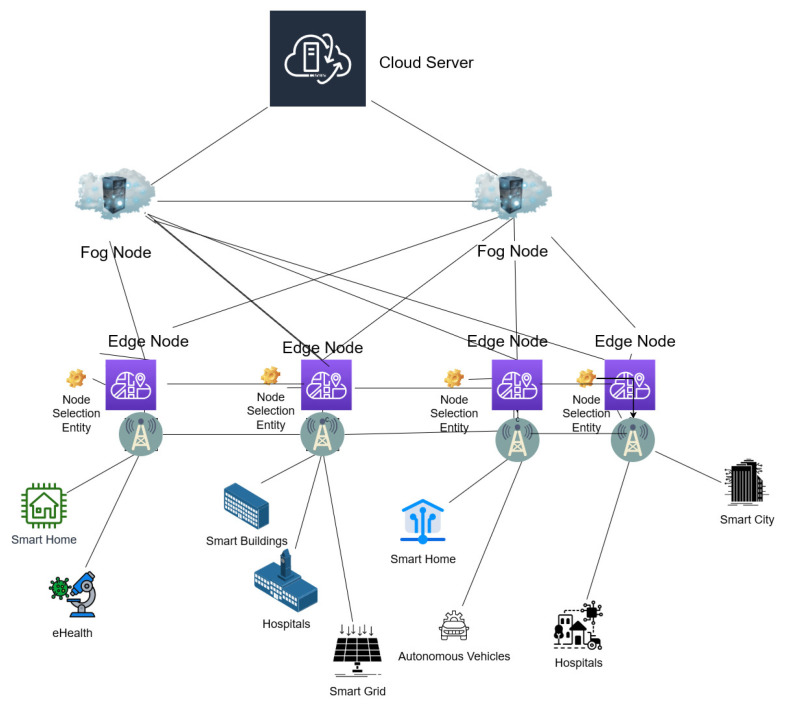
IoT network architecture.

Our proposal takes into account a comprehensive IoT network architecture that encompasses edge, fog, and cloud as computation layers to cater for diverse, advanced IoT applications. The IoT network architecture considered is illustrated in Figure 2. In this architecture, all the nodes at three layers have been deployed with a varying number of servers with variable resource capacity. At the top of the architecture is the cloud layer, responsible for processing IoT requests with minimal QoS requirements. The middle tier is the fog layer, consisting of fog nodes that perform collation, processing, and data analysis for advanced IoT use cases such as smart cities, smart grids, and autonomous vehicles. The final layer is the edge layer, comprising base stations that serve as edge servers for faster processing of IoT use cases that have strict QoS requirements, including low latency. The edge servers have been deployed closer to the end user for quick access to IoT requests. For faster and quick processing of most of the IoT requests, we have considered that each node at fog and edge layers are deployed with a custom number of servers. To achieve energy-efficient operation, an optimal node selection entity can be designed and deployed at the edge layer to select and process each IoT request from different IoT devices, taking advantage of all three layers of the IoT network architecture. The node selection entity would be designed to verify that the application-specific QoS and IoT architecture-specific network constraints are met for each IoT request. Our optimal node selection framework considers the energy consumption of different components at all three layers. For optimized energy management, the costs of energy consumption involved in activating and running the servers/nodes at fog and edge layer are considered. Further, a steady energy cost of processing the IoT request at cloud layer is considered because the actual cost of processing a request at cloud layer would be complex to calculate due to the complex implementation of the cloud architecture. The cost component for each of the layers is derived from [39]. Once we run the optimal node selection framework, the IoT request will be directed to an optimally selected node for processing. In the next subsection, we elaborate on the mathematical model used in our proposed optimization framework for optimal node selection. This framework is an extension of the framework introduced in [24], which lacked the flexibility of the number of servers deployed at each node and consideration of energy component in the optimization framework. The limitation of our framework is that we have not considered mobility of the IoT request after it has been received at a particular node at the edge layer. Hence our framework emphasizes the fact that once an IoT request is received at a particular node at the edge layer, the IoT request is steady and cannot be mobile or move around to different nodes at the edge layer while it is being processed.

### 4.1. Optimization Framework

It is crucial to have an optimal node selection mechanism for optimal resource handling and efficient energy management in IoT networks with diverse use cases. The proposed framework aims to minimize the overall energy usage of the entire IoT architecture when processing all the IoT requests received at the edge layer within a predetermined time frame. It takes into account the varying number of servers with different capacities deployed at each node in the edge-fog-cloud layer architecture. We have also considered a finite energy cost utilized for running a single server at each node in edge-fog-cloud layers [39]. A finite energy cost is also defined and considered for running a node at the fog and edge layer [39].

Our framework also considers active and inactive nodes. Active nodes mean the nodes are currently active and are being used. The inactive node denotes that the node is connected to the network, but none of its servers are activated or running. Making unused computation nodes inactive, the energy consumption in the entire IoT architecture can be reduced [24]. Moreover, the mechanism of activation and running of a node and server is controlled by a defined cost involved in activating and running the node and the server and various QoS metrics. With the usage of cost factor and QoS requirements like resource availability and latency, our optimization framework balances the trade-off for processing the IoT request between activation of an inactive node against an already active node. In addition, the framework simultaneously balances the trade-off between usage of a server at an already active node against usage of a server at an inactive node for processing the IoT request.

The objective of the frameworks is to minimize the energy cost utilized for processing the incoming IoT request at all three layers. In addition, the framework also ensures the satisfaction of each and every demand of (1) IoT applications and their use cases, like delay, bandwidth, latency, and resource processing capacity and (2) communication architecture, like bandwidth availability, delay supported, and connectivity, are satisfied. Integer Linear Programming (ILP) is used for developing the node selection optimization framework. ILP-based optimisation frameworks were widely used in network optimisations [40,41]. The next subsection clearly explains different sets, various parameters, and variables defined and used in the proposed node selection optimization framework. We further emphasize the proposed objective function and its respective constraints.

### 4.2. Parameters and Sets

We incorporate diverse sets and parameters to depict the requirements of various IoT applications and data related to networks, computing nodes, their locations, and connectivity.

#### 4.2.1. Sets

- Let E = 1,…, $n_{E}$: denotes a set of all nodes to be considered the edge layer
- Let F = 1,…, $n_{F}$: denotes set of all nodes to be considered at the fog layer
- Let C = 1,…, $n_{C}$: denotes set of all nodes to be considered at the cloud layer
- Let $L_{o}=1,\ldots ,n_{L}$: denote the set of all the nodes collectively at all three layers, Lo=E∪F∪C
- Let $E_{s}=1,\ldots ,s_{e}$: denote set of edge servers
- Let $F_{s}=1,\ldots ,s_{f}$: denote set of fog servers
- Let $C_{s}=1,\ldots ,s_{c}$: denote set of cloud servers
- Let jobn: 1,…,job List all IoT requests/jobs from various IoT use cases

#### 4.2.2. Network Parameters

- $n_{C}$: The overall count of nodes installed at the cloud layer.
- $n_{F}$: The overall count of nodes installed at the fog layer
- $n_{E}$: The overall count of nodes installed at the edge layer
- $n_{L}$: The overall count of nodes installed in the IoT network
- $s_{e}$: Maximum number of edge servers that can be deployed at single edge node
- $s_{f}$: Maximum number of fog servers that can be deployed at single fog node
- $s_{c}$: Maximum number of cloud servers that can be deployed at single cloud node
- $L_{e}\left[l\right]$: Parameter denoting location l where an edge node e is installed
- $L_{f}\left[l\right]$: Parameter denoting location l where a fog node f is installed
- $L_{c}\left[l\right]$: Parameter denoting location l where a cloud node c is installed
- L[l]: Parameter denoting location l where a node has been deployed in the IoT network graph
- $N_{e}\left[E\right]$: Parameter denoting the number of servers installed at every node in edge layer
- $N_{f}\left[F\right]$: Parameter denoting the number of servers installed at every node in fog layer
- $N_{c}\left[C\right]$: Parameter denoting the number of servers installed at every node in cloud layer
- $NE_{si}\left[x\right]\left[y\right]$: Boolean parameter, $NE_{si}\left[x\right]\left[y\right]=1$ if $x^{th}$ nodes has an initially deployed active server at $y^{th}$ position, NEsi[x][y]=0 otherwise, where $y\in E_{s}$ and x∈E
- $NF_{si}\left[x\right]\left[y\right]$: Boolean parameter, $NF_{si}\left[x\right]\left[y\right]=1$ if $x^{th}$ nodes has an initially deployed active server at $y^{th}$ position, $NF_{si}\left[x\right]\left[y\right]=0$ otherwise, where y∈Fs and x∈F
- $NC_{si}\left[x\right]\left[y\right]$: Boolean parameter, $NC_{si}\left[x\right]\left[y\right]=1$ if $x^{th}$ nodes has an initially deployed active server at $y^{th}$ position, $NC_{si}\left[x\right]\left[y\right]=0$ otherwise, where y∈Cs and x∈C
- $R_{en}\left[l\right]$: Remaining resource/processing capacity at the specific location denoted by *l*, of an edge node
- $R_{fn}\left[l\right]$: Remaining resource/processing capacity at the specific location denoted by *l*, of a fog node
- $R_{cn}\left[l\right]$: Remaining resource/processing capacity at the specific location denoted by *l*, of a cloud node
- $R_{es}\left[l\right]$: denotes the resource/processing capacity of each server at an edge node in the network at location *l*
- $R_{fs}\left[l\right]$: denotes the resource/processing capacity of each server at a fog node in the network *l*
- $R_{cs}\left[l\right]$: denotes the resource/processing capacity of each server at a cloud node in the network location *l*
- $B{}_{e}\left[l\right]$: Bandwidth supported for communication at the specific location denoted by *l*, of an edge node
- $B{}_{f}\left[l\right]$: Bandwidth supported for communication at the specific location denoted by *l*, of an fog node
- $B{}_{c}\left[l\right]$: Bandwidth supported for communication at the specific location denoted by *l*, of an cloud node
- d[x][y]: Total delay between the $x^{th}$ node and $y^{th}$ node in the IoT network
- g[x][y]: Boolean parameter denoting connectivity among nodes in the IoT network architecture, g[x][y] =1 on a condition that connectivity exists among $x^{th}$ node and $y^{th}$ node else 0
- $C_{en}$: Cost of activating an inactive E node for processing
- $C_{fn}$:Cost of activating an inactive F node for processing
- $C_{es}$: Cost of activating an inactive server at edge node
- $C_{fs}$: Cost of activating an inactive server at fog node
- $C_{c}$: Cost of running a job at cloud

#### 4.2.3. IoT Application Job Request Parameters

- job: denotes the maximum count of IoT request/jobs
- $j{}_{r}$: denotes resource/processing requirement of an IoT request
- $j{}_{b}$: denotes bandwidth requirement of an IoT request
- $j{}_{l}$: denotes latency requirement of an IoT request
- $j{}_{o}$: denotes origin node of an IoT request

### 4.3. Variables

- e[j][e]: A Boolean variable, takes the value 1 if the $j^{th}$ job is assigned to the $e^{th}$ edge node, and 0 otherwise, where e∈E and j∈jobn
- f[j][f]: is 1 if $j^{th}$ job is assigned to $f^{th}$ fog node, and 0 otherwise, where f∈F and j∈jobn. This is a Boolean variable.
- c[j][c]: is 1 if $j^{th}$ job is assigned to $c^{th}$ cloud node, and 0 otherwise, where c∈C and j∈jobn. This is a Boolean variable.
- $E{}_{a}$[*e*]: is $E{}_{a}$[*e*] = 1 if $e^{th}$ is an active edge node, $E{}_{a}$[*e*] = 0 otherwise where e∈E. This is a Boolean variable that represents current active edge nodes.
- $F{}_{a}$[*f*]: is $F{}_{a}$[*f*] = 1 if $f^{th}$ is an active fog node, $F{}_{a}$[*f*] = 0 otherwise where f∈F. This is a Boolean variable that represents current active fog nodes.
- $C{}_{a}$[*c*]: is $C{}_{a}$[*c*] = 1 if $c^{th}$ is an active cloud node, $C{}_{a}$[*c*] = 0 otherwise where c∈C. This is a Boolean variable that represents current active cloud nodes.
- $NE_{s}\left[e\right]\left[x\right]$: Boolean variable, $NE_{s}\left[e\right]\left[x\right]=1$ if $e^{th}$ nodes has an active server at $x^{th}$ position, $NE_{s}\left[e\right]\left[x\right]=0$ otherwise, where x∈Es and e∈E
- $NF_{s}\left[f\right]\left[x\right]$: Boolean variable, $NF_{s}\left[f\right]\left[x\right]=1$ if $f^{th}$ nodes has an active server at $x^{th}$ position, $NF_{s}\left[f\right]\left[x\right]=0$ otherwise, where x∈Fs and f∈F
- $NC_{s}\left[c\right]\left[x\right]$: Boolean variable, $NC_{s}\left[c\right]\left[x\right]=1$ if $c^{th}$ nodes has an active server at $x^{th}$ position, $NC_{s}\left[c\right]\left[f\right]=0$ otherwise, where x∈Cs and c∈C

### 4.4. Objective Function of the Framework

The framework aims to minimize the objective function, which is the cost of energy utilized for processing IoT requests in the IoT network architecture. The framework’s objective function, as stated in Equation (1), aims to minimize overall energy cost across the edge, fog, and cloud layers, when processing new IoT requests. The objective function considers the cost of activating a new node at fog and edge layers, $C_{fn}$ and $C_{en}$, respectively. The cost function also considers the cost of activating a server at an active node in fog and edge layers, $C_{fs}$ and $C_{es}$, respectively. Further, it also consists of the cost of running a job at the cloud layer, as the nodes will always be active.

(1) $$\begin{array}{c} \;Minimize(\sum_{x\epsilon E}\left(E{}_{a}\left[x\right]-L_{e}\left[x\right]\right)*C_{en}+\\ \sum_{x\epsilon E}\sum_{s\epsilon Es}\left(NE{}_{s}\left[x\right]\left[s\right]-NE_{si}\left[x\right]\left[s\right]\right)*C_{es}+\\ \sum_{y\epsilon F}\left(F{}_{a}\left[y\right]-L_{f}\left[y\right]\right)*C_{fn}+\\ \sum_{x\epsilon F}\sum_{s\epsilon Fs}\left(NF{}_{s}\left[x\right]\left[s\right]-NF_{si}\left[x\right]\left[s\right]\right)*C_{fs}+\\ \;\sum_{z\epsilon C}\left(C{}_{a}\left[z\right]-L_{c}\left[z\right]\right)*C_{c}) \end{array}$$

### 4.5. Constraints

The framework minimizes the cost of energy used for processing the IoT request whilst satisfying the network and IoT application use case demands. This subsection presents the constraints that pertain to these requirements and demands.

#### 4.5.1. Network Constraints

- In the IoT architecture, allocating a new job/request to a node must be restricted so that each IoT job is processed solely at a single node. This constraint is denoted in Equation (2) wherein the variables e,f,j are used to correspondingly record the values of every job assigned to each edge-fog-cloud location.
  (2) $$\sum_{x\epsilon E}e\left[j\right]\left[x\right]+\sum_{y\epsilon F}f\left[j\right]\left[y\right]+\sum_{z\epsilon C}c\left[j\right]\left[z\right]=1,\forall \;j\;in\;jobn$$
- To restrict the overall count of currently active nodes in the IoT network architecture, we have defined a constraint in our framework to ensure that the count of active nodes does not exceed the total count of nodes deployed at each layer. This is highlighted in Equations (3)–(5), where the maximum allowable number of nodes at each layer are denoted by nE,NC,nF and active nodes are denoted by $E{}_{a},F{}_{a},C{}_{a}$ at edge, fog, and cloud layers, respectively.
  (3) $$\sum_{l\epsilon E}E{}_{a}\left[l\right]\leq n_{E}$$
  (4) $$\sum_{l\epsilon F}F{}_{a}\left[l\right]\leq n_{F}$$
  (5) $$\sum_{l\epsilon C}C{}_{a}\left[l\right]\leq n_{C}$$
- A constraint is required to restrict the count of servers activated at each node, ensuring that the number does not exceed the total count of servers installed at the corresponding layer. This constraint is defined in Equations (6)–(8) where $N{}_{e},N{}_{f},N{}_{c}$ are the total count of servers installed at each node at edge, fog, and cloud layers, respectively.
  (6) $$\sum_{s\epsilon E_{s}}NE{}_{s}\left[l\right]\left[s\right]\leq s{}_{e}\left[l\right],\forall \;l\in E$$
  (7) $$\sum_{s\epsilon F_{s}}NF{}_{s}\left[l\right]\left[s\right]\leq s{}_{f}\left[l\right],\forall \;l\in F$$
  (8) $$\sum_{s\epsilon C_{s}}NC{}_{s}\left[l\right]\left[s\right]\leq s{}_{c}\left[l\right],\forall \;l\in C$$

#### 4.5.2. Application Constraints

- For verifying the delay requirement for IoT use cases and the network connectivity among the nodes where the request has originated and the optimal node is selected for processing the request, a constraint has been defined using Equations (9)–(11). The delay and the network connectivity for all the layers cannot exceed the latency of the IoT job request.
  (9) $$\begin{array}{l} e\left[j\right]\left[a\right]*(g\left[j{}_{o}\left[j\right]\right]\left[a+n_{C}+n_{F}\right]*\left(d\left[j{}_{o}\left[j\right]\right]\left[a+n_{C}+n_{F}\right]\right)\\ <=j{}_{l}\left[j\right],\forall \;j\;in\;jobn,\forall \;a\;in\;E \end{array}$$
  (10) $$\begin{array}{c} f\left[j\right]\left[a\right]*(g\left[j{}_{o}\left[j\right]\right]\left[a+n_{C}\right]*\left(d\left[j{}_{o}\left[j\right]\right]\left[a+n_{C}\right]\right)\\ <=j{}_{l}\left[j\right],\forall \;j\;in\;jobn,\forall \;a\;in\;F \end{array}$$
  (11) $$\begin{array}{c} c\left[j\right]\left[a\right]*(g\left[j{}_{o}\left[j\right]\right]\left[a\right]*\left(d\left[j{}_{o}\left[j\right]\right]\left[a\right]\right)<=j{}_{l}\left[j\right],\forall \;j\;in\;\\ jobn,\forall \;a\;in\;C \end{array}$$
- For each request allocated to a node, it is essential to ensure that the allocated node has enough leftover capacity for processing the job, whether at the edge, fog, or cloud layer. The remaining capacity at each node at edge-fog-cloud layer is denoted by Ren,Rfn,Rcn. The Equations (12)–(14) assist in upholding the constraint by verifying that the difference between the resource capacity of a node and the resource requirement of a job is non-negative across all three layers.
  (12) $$R_{es}\left[l\right]*Ne\left[l\right]-R_{en}\left[l\right]-\sum_{j\epsilon jobn}e\left[j\right]\left[l\right]*j{}_{r}\left[j\right]\geq 0,\forall \;l\;in\;E$$
  (13) $$R_{fs}\left[l\right]*Nf\left[l\right]-R_{fn}\left[l\right]-\sum_{j\epsilon jobn}f\left[j\right]\left[l\right]*j{}_{r}\left[j\right]\geq 0,\forall \;l\;in\;F$$
  (14) $$R_{cs}\left[l\right]*Nc\left[l\right]-R_{cn}\left[l\right]-\sum_{j\epsilon jobn}c\left[j\right]\left[l\right]*j{}_{r}\left[j\right]\geq 0,\forall \;l\;in\;C$$
- We formulate a constraint to ensure that the available bandwidth supported by the node is adequate to satisfy the job’s bandwidth requirement, considering the count of servers installed at each node in the edge-fog-cloud layer. It is defined in Equations (15)–(17), respectively. To adhere to the constraint, we subtract the job’s required bandwidth from the node’s supported bandwidth and ensure that the result is always a positive integer at the edge-fog-cloud layer.
  (15) $$B{}_{e}\left[l\right]-\sum_{j\epsilon jobn}e\left[j\right]\left[l\right]*j{}_{b}\left[j\right]\geq 0,\forall \;l\;in\;E$$
  (16) $$B{}_{f}\left[l\right]-\sum_{j\epsilon jobn}f\left[j\right]\left[l\right]*j{}_{b}\left[j\right]\geq 0,\forall \;l\;in\;F$$
  (17) $$B{}_{c}\left[l\right]-\sum_{j\epsilon jobn}c\left[j\right]\left[l\right]*j{}_{b}\left[j\right]\geq 0,\forall \;l\;in\;C$$

#### 4.5.3. Limits

- To ensure that the number of jobs allocated to a location at each layer does not exceed the number of activated nodes at that layer, a constraint is required. Equations (18)–(20) are used to define this constraint in the edge, fog, and cloud layers. This constraint guarantees that when an IoT request is assigned to either of the nodes e,f,c, the corresponding nodes $E_{a}$, $F_{a}$, $C_{a}$ are active.
  (18) $$e\left[j\right]\left[l\right]\leq E_{a}\left[l\right],\forall \;j\;in\;jobn\;\forall \;l\;in\;E$$
  (19) $$f\left[j\right]\left[l\right]\leq F_{a}\left[l\right],\forall \;j\;in\;jobn\;\forall \;l\;in\;F$$
  (20) $$c\left[j\right]\left[l\right]\leq C_{a}\left[l\right],\forall \;j\;in\;jobn\;\forall \;l\;in\;C$$
- A constraint is necessary to guarantee that while processing a new IoT request, the total count of nodes being activated at a specific layer is greater than or equal to the total count of activated nodes installed initially. Equations (21)–(23) are used to define this constraint in the edge, fog, and cloud layers. These equations ensure that the total count of activated nodes in each layer, denoted by $E_{a}$, $F_{a}$, and $C_{a}$, respectively, can be equal to or greater than the total count of nodes initially deployed and activated at the respective layer
  (21) $$E{}_{a}\left[l\right]\geq L{}_{e}\left[l\right],\forall \;l\;in\;E$$
  (22) $$F{}_{a}\left[l\right]\geq L{}_{f}\left[l\right],\forall \;l\;in\;F$$
  (23) $$C{}_{a}\left[l\right]\geq L{}_{c}\left[l\right],\forall \;l\;in\;C$$
- A constraint is necessary to ensure that the total count of servers being activated at each node at a specific layer for processing the incoming IoT job requests does not exceed the total count of nodes already active at the respective layer. Equations (24)–(26) are used to define this constraint in the edge, fog, and cloud layers, ensuring that the activated servers at the edge ($NE_{s}$), fog ($NF_{s}$), and cloud layer ($NC_{s}$) cannot exceed the count of nodes already installed and active at each layer.
  (24) $$NE{}_{s}\left[l\right]\left[s\right]\leq E{}_{a}\left[l\right],\forall \;l\;in\;E,\forall \;s\;in\;E_{s}$$
  (25) $$NF{}_{s}\left[l\right]\left[s\right]\leq F{}_{a}\left[l\right],\forall \;l\;in\;F,\forall \;s\;in\;F_{s}$$
  (26) $$NC{}_{s}\left[l\right]\left[s\right]\leq C{}_{a}\left[l\right],\forall \;l\;in\;C,\forall \;s\;in\;C_{s}$$
- To ensure that the number of servers activated at each node in a layer for processing the incoming IoT requests is not greater than the number of nodes already active at that location, a constraint is required. Equations (27)–(29) make sure the constraint is satisfied where the server activated at the edge ($NE{}_{s}$), fog ($NF{}_{s}$), and cloud ($NC{}_{s}$) layer can be greater than or equal to the number of active servers during the initial deployment $NE{}_{si},NF{}_{si},\;\mathrm{and}\;NC{}_{si}$, respectively.
  (27) $$NE{}_{s}\left[l\right]\left[s\right]\geq NE{}_{si}\left[l\right]\left[s\right],\forall \;l\;in\;E,\forall \;s\;in\;E_{s}$$
  (28) $$NF{}_{s}\left[l\right]\left[s\right]\geq NF{}_{si}\left[l\right]\left[s\right],\forall \;l\;in\;F,\forall \;s\;in\;F_{s}$$
  (29) $$NC{}_{s}\left[l\right]\left[s\right]\geq NC{}_{si}\left[l\right]\left[s\right],\forall \;l\;in\;C,\forall \;s\;in\;C_{s}$$

## 5. Framework Evaluation

**Figure 3:**
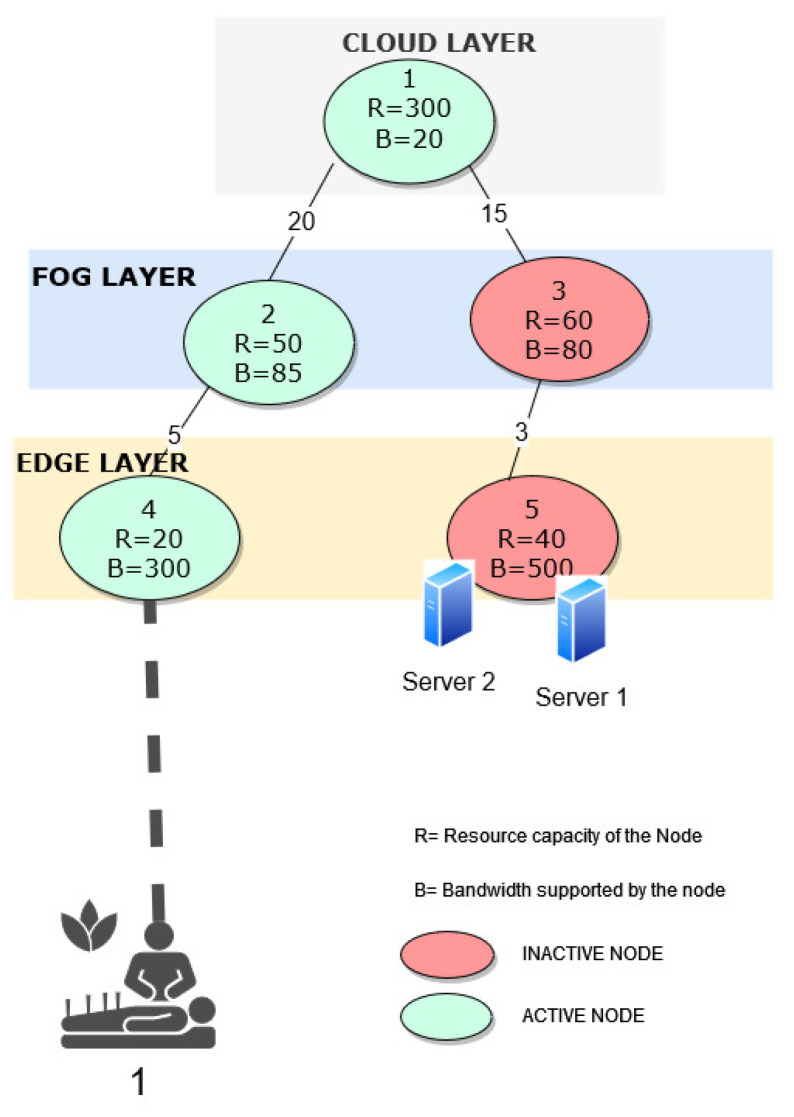
Sample network graph for multi-layered IoT architecture.

In this section, we assess the framework under diverse configurations. The proposed framework is evaluated using IBM CPLEX. Throughout this section, we use network graphs to present the data sets and optimal solutions of our framework. Therefore, we first use a sample network graph shown in Figure 3 to explain the notations used in the network graphs. As shown in the sample network graph, different nodes are deployed at cloud, fog, and edge layers.

The network graph has active and inactive nodes. An active node is a node that is available for computation. Though some of the nodes are inactive, the communication links that connect all the nodes are always active. In the graphs, active nodes are denoted in green circles, and inactive nodes are denoted in red circles. Each node’s supported resource capacity and bandwidth are represented by R and B, respectively. Each node is numbered for easy identification. Most of the nodes are deployed with a single server. However, the nodes deployed with more than one server are also represented in the graph. For example, Node 5 has two servers, and therefore, Node 5 is attached to two blue server icons in Figure 3. The direct connectivity between the nodes at all the layers is represented by a solid line. The number on the black solid line represents the delay of the corresponding link.

In the network graph, the incoming IoT requests are illustrated with different icons representing the type of IoT request. Each IoT request is numbered and connected to a node at which they were received or processed with a dotted line. For example, in Figure 3, the IoT request 1, which is an eHealth application, is received at Node 4 in the edge layer. For the evaluation, we consider requests from diverse IoT applications, like smart health, smart city/grid, smart vehicles, and smart factories.

Next, we provide detailed analyses of each experiment and scenarios that we consider for the framework evaluation and their outcome.

### 5.1. Optimal Solutions of Experiment 1

**Figure 4:**
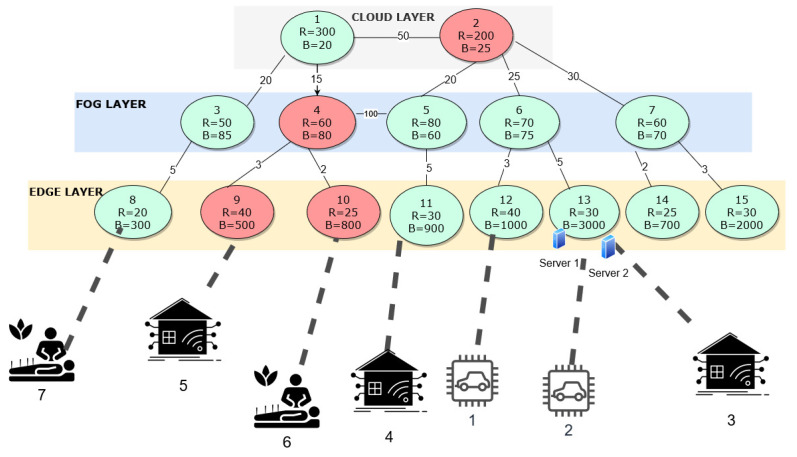
Network configuration of Experiment 1.

Figure 4 illustrates the network data set we consider for experiment 1. The data set consists of eight edge, five fog, and two cloud nodes deployed at respective layers. The node number, their respective processing capacity, and the bandwidth supported by the respective node have been highlighted in Figure 4. There is an energy cost for running each node and a cost for running a server at each node at the edge and fog layers. We only consider the cost of executing a job in the cloud, as the cloud nodes will always be active. The energy costs of activating and running the node and the servers have been defined using [39]. The energy cost has been normalized for result analysis and a graphical representation. The cost considered at each node and at each layer, is the cost of running the switch, the router and the server. The normalized cost is one tenth of the actual cost incurred at each node at edge, fog and cloud layer. The cost of running the edge and fog servers at each node is defined as 50 and 30, respectively. The cost of activating an edge and fog node is 20 and 50, respectively. The cost for executing the job at the cloud node is 90. The inter-connectivity and latency between the nodes are computed beforehand and saved in the parameters *g* and *d*, respectively. Please note that the energy cost values presented in the rest of the paper are the normalized energy cost values.

The network nodes are deployed in various locations and have varying resource capacities and bandwidth to be supported. A varying number of servers are also deployed at each node location. We consider various IoT requests with varying requirements for evaluation. Each new incoming request is associated with several parameters such as its computation resource ($j_{r}$), bandwidth ($j_{b}$), and latency ($j_{l}$) requirements, as well as its origin node ($j_{o}$). As a result, each new request identified by a unique job/request number *j* can be described as “Job $j[j{}_{r},j{}_{b},j{}_{l},j{}_{o}]$”. The framework is evaluated using four distinct scenarios to examine its performance under various conditions and request parameters.

**Figure 5:**
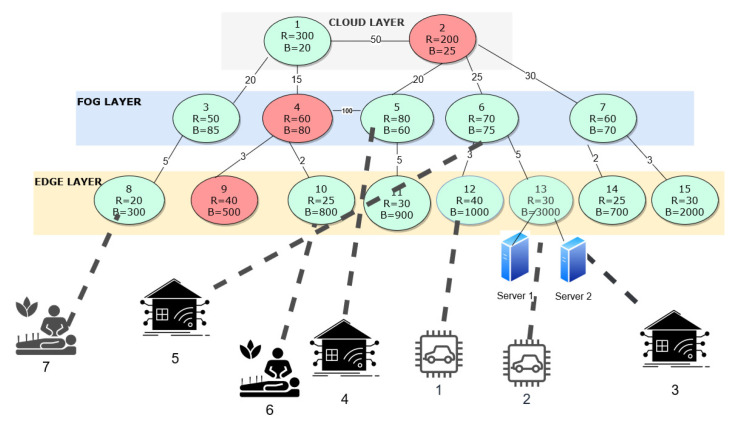
Graphical illustration of the optimal solution for Scenario 4.

In the initial scenario, we have deactivated node 2 from the cloud layer, node 4 from the fog layer, and nodes 9 and 10 from the edge layer, leading to 11 active nodes in our IoT network for computation, with one server deployed at each node. None of the servers are active during initial deployment, as shown in Figure 4. In this scenario, to assess the effectiveness of our optimization framework, we consider the case where a low-latency IoT request originated at edge node 12 and is labeled as Job 1[40, 1000, 10, 12]. Upon applying our framework to this request, we find that the job can be assigned to the existing active node 12 at the edge layer without activating any further nodes in the IoT network and activating only a single server at edge node 12. The total amount of energy used to process that request is 50. As can be seen in the optimal solution, a single server in Node 12 is activated to process the request over activating an additional node to process the request. The dotted lines in Figure 5 represent the chosen node for processing a specific IoT request.

For the second scenario, we consider the same graph as in the above scenario with an increasing number of incoming IoT job requests. We have three IoT job requests originating from eHealth, autonomous vehicles, and smart grid applications with varying requirements, and they are represented as Job 1[40, 1000, 10, 12], Job 2[30, 1100, 6, 13], and Job 3[30, 1100, 65, 13], respectively. The optimal allocation for these three jobs was obtained using the proposed optimization framework. The optimal allocation is shown in Figure 5. Jobs 1, 2, and 3 are allocated to edge nodes 12, 13, and 15, respectively, with one server being activated at the respective locations. The optimal cost of energy used for processing the three requests is 150. The results show that the total count of active nodes stayed unchanged, indicating that the framework ensures the processing of the requests with minimal utilization of resources, nodes, servers, and energy.

For the third scenario, the IoT network graph maintained from scenario 2, with 11 active nodes, was utilized but with an increased number of servers at edge node 13 to two and the rest of the nodes with a single server each. The received IoT jobs were the same as in scenario 2, and we utilized our optimization framework to obtain the best allocation for the jobs. As shown in Figure 5, jobs 1, 2, and 3 were assigned to node 12, node 13, and node 13, all at the edge layer, respectively, with one server activated at edge node 12 and both the servers activated at edge node 13, respectively. Moreover, the minimum energy cost used to process the request was 150, which was the optimal solution. Based on the result, we can conclude that the number of active nodes remained constant in scenario 3, indicating that the framework was effective in minimizing the use of servers, nodes, and energy while processing all the received requests efficiently. Thus with the two servers deployed at edge node 13 and for processing the IoT requests, the optimization framework, balanced the trade-off- between activation of new node against usage of the node which was already active and also the trade-off between activating a new server at an already active node against activating the server at an inactive node.

In the fourth scenario, we utilize the IoT network graph described in Figure 4 with 11 active nodes, 2 servers deployed at edge node 13, and a single server deployed at all other nodes. In this scenario, 7 different jobs with specific requirements were received, labeled as Job 1 to Job 7, each with varying amounts of data, latency requirements, and destination nodes. Using the optimization framework, the best allocation for jobs (from 1 to 7) was determined. The optimal allocation framework assigned Job 1 to edge node 12, Job 2 and 3 to edge node 13, Job 4 to fog node 5, Job 5 to fog node 6, Job 6 to edge node 10, and Job 7 to edge node 8. The allocation was based on the framework’s optimization of resource utilization, such as servers, nodes, and energy. The results show that the framework was capable of processing different types of jobs with diverse requirements while using minimal resources. The allocation is visualized in Figure 5, with two servers activated at edge node 13 and a single server activated at each respective node where the job was processed. Further, the minimum energy cost incurred in processing the request was 330, which is the optimal solution. The outcome demonstrates that the number of active nodes remained constant, implying that the framework effectively processes all IoT requests while minimizing the use of servers, nodes, and energy.

**Figure 6:**
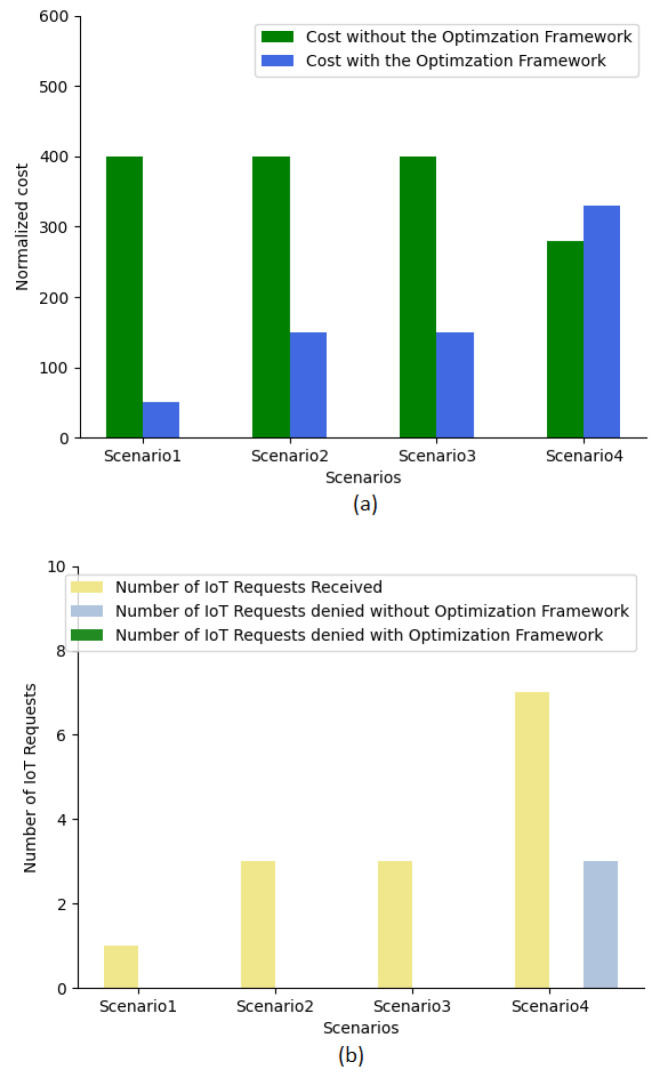
(**a**) Energy cost comparison with and without optimization (**b**) Comparison of IoT request received and denied without usage of optimization, for Scenarios 1, 2, 3, and 4.

We have compared the optimal energy cost for scenarios when the optimization framework was not used to conduct the node selection. When the optimization framework was not used, the job was allocated to its nearest node and assuming all the nodes were active and were deployed with a single server. Figure 6 illustrates the comparison results of scenarios 1,2, 3, and 4. Figure 6a shows the energy cost comparison, and Figure 6b indicates the total IoT requests received and the range of IoT requests denied in all scenarios. As we can see from Figure 6a, for processing different IoT requests with the usage of the optimization framework, the energy costs incurred for scenarios 1, 2, and 3 were significantly low, compared to the energy cost incurred for processing IoT request without the optimization framework. Further, in scenario 4, the energy cost incurred to process all the seven IoT requests with optimization was slightly high in comparison to those without optimization framework. This was mainly because, when the optimization framework was not used, three IoT requests were denied, as shown in Figure 6b. When the optimization framework was used, all the requests were processed by allocating them to available nodes in the edge-fog-cloud layer that can satisfy the application requirements.

### 5.2. Optimal Solutions of Experiment 2

**Figure 7:**
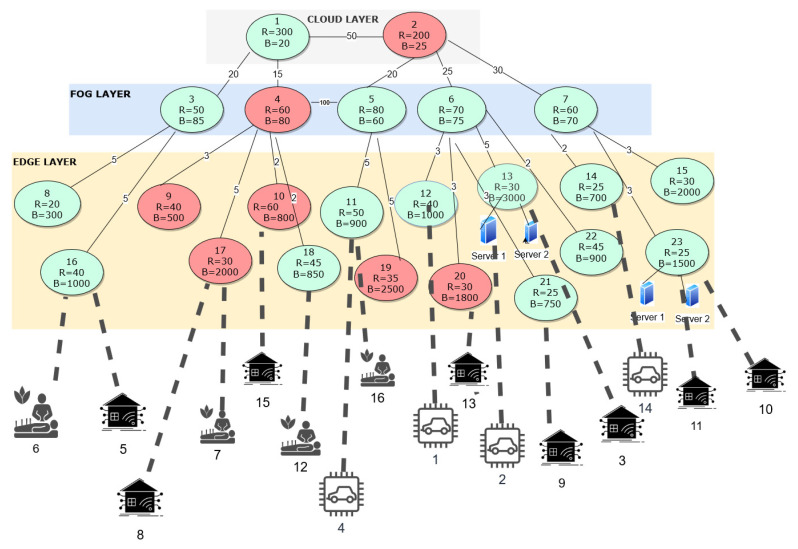
Network configuration of Experiment 2.

In experiment 2, we have considered a more complex network data set with 2 cloud nodes, 5 fog nodes, and 16 edge nodes. The data set is illustrated in Figure 7. The energy cost has been normalized for result analysis and a graphical representation. The cost of running the edge and fog servers at each node is defined as 60 and 30, respectively. The cost of activating an edge and fog node is 80 and 50, respectively. The cost for executing the job at the cloud node is 90. We evaluated this data set under five scenarios ranging from scenario 0 to scenario 4. These five scenarios illustrate different conditions of the IoT architecture, cost values, and the frameworks used to obtain the allocations.

#### 5.2.1. Scenario 0: Optimal Solution

**Figure 8:**
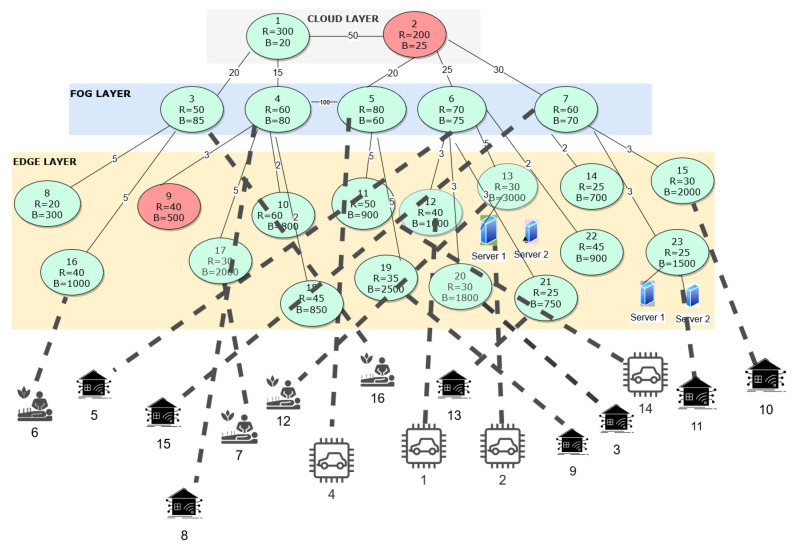
Graphical illustration of the optimal solution.

In this scenario, we have considered a three-layered IoT architecture, and all the nodes in the IoT architecture have limited resources and connectivity/latency between the nodes. Each new IoT request received by the IoT architecture is defined with a job number and is illustrated in Table 3. All the IoT requests received at the edge layer are from different IoT use cases like eHealth, autonomous vehicles, smart grids, and smart factories. We have considered that we run our proposed optimization framework with normal cost derived after analyzing the actual power consumption involved in running the edge and fog layer devices. The incoming 16 jobs are shown in Table 3. The outcome after running the optimization framework is shown in Figure 8 with dotted lines, which highlights the allocation of each job/IoT request at the respective node capable of processing the respective IoT request at either of the layers. The resulting minimized cost after running the optimization framework was 1100. The optimally allocated nodes for processing each IoT request with custom requirements is also shown in Table 3. This was the optimal solution for the complex data set using our optimization framework. If we were to run the above optimization framework for the same set of IoT requests but with an assumption that all the nodes were active and have unlimited resources and there is not any limitation on connectivity/latency, then all the nodes at the edge layer would be utilized to process all the 16 IoT requests and the cost incurred in processing all the IoT requests would be 1330.

#### 5.2.2. Scenario 1: Cost Sensitivity

**Figure 9:**
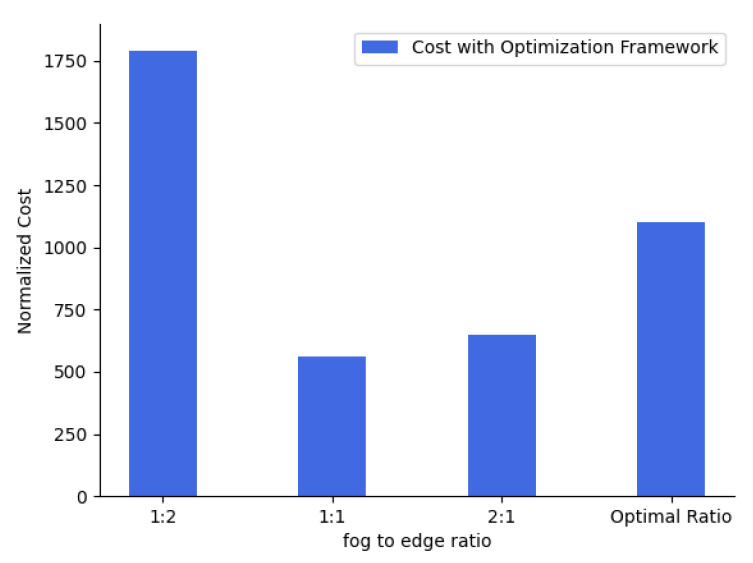
Cost sensitivity Analysis for Fog and Edge layer.

We are aware that the cost of deployment of the cloud layer is too high, and it is not really flexible to manage the nodes at the cloud layer. Hence, in this scenario, we have emphasized the fact that when the cost of the edge layer or fog layer (usage and deployment) is too high, then how the optimization framework performs, allocates the jobs, and how the overall cost is affected. Hence, we have compared the cost of running the edge-cloud layer against the fog-cloud layer using their ratios for our considered IoT architecture with the optimization framework. We ran the optimization framework for the same set of 16 jobs as shown in Table 3 but with the cost of the fog-to-edge layer ratio as 1:2 and produced the optimization results. The resultant cost after optimization was 1790, wherein the framework activated the maximum possible fog nodes and the least amount of edge nodes to meet their requirements. Hence we have used 5 fog nodes and 10 edge nodes with a single server activated and utilized at each node. We re-run the framework for the same set of 16 jobs as shown in Table 3, but now the ratio of fog to edge layer was 2:1. The resultant cost after optimization goes down, as most of the IoT requests were being processed at an edge node and the optimization framework activated the least amount of fog nodes. Hence we have incurred a total cost of 650 and have used 3 fog nodes and 12 edge nodes with a server activated and used at each node. Figure 9 represents the comparison of the cost sensitivity between the fog layer and edge layer when the cost ratio was 1:2, 1:1, 2:1, and optimal ratio, respectively. It can be observed from Figure 9 that when the cost ratio of the fog-to-edge layer was optimal, the cost incurred in processing the IoT requests using our optimization framework was balanced, and there was optimal utilization of IoT architecture resources. However, when the cost ratio of the fog-to-edge layer was 1:1 and 2:1, the cost incurred in processing the IoT requests was low, the IoT architecture resources was over-utilized at either of the layers, which would not be beneficial in the long run for the IoT architecture. Hence, we can draw the conclusion that there should be a balance between the deployment and utilization of the fog layer and edge layer, and not all IoT requests can be processed by increasing the number of nodes at either the fog or edge layers, ideally.

#### 5.2.3. Scenario 2: Single Layer Deployed (Either Edge/Fog Layer)

**Figure 10:**
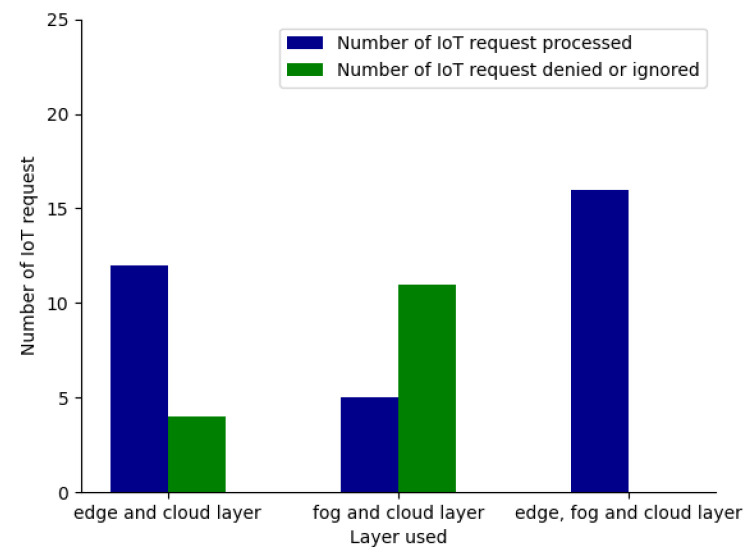
Efficiency of request processing against the sensitivity of the layer deployed.

**Figure 11:**
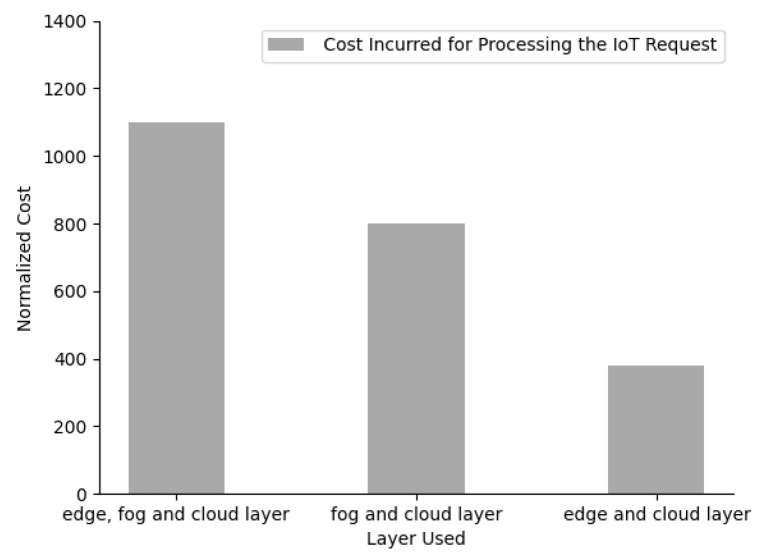
Sensitivity of layer deployed against cost.

With the layered IoT architecture initially being considered, we have focused on the deployment of only two layers which are only fog and cloud layer and no edge layer. So when the same 16 IoT requests described in Table 3 are considered, only 5 IoT request numbers 4, 5, 8, 15, and 16 would be processed, and the rest of the 10 IoT requests would be ignored by our optimization framework, as the IoT architecture and the framework will not be able to meet their requirements. Similarly, later, we considered the deployment of only two layers which were the edge layer and the cloud layer, and there was no fog layer in our architecture. With those same 16 IoT requests being received by our IoT architecture, only 13 IoT requests would be processed, and the other 3 IoT request numbers 4, 5, and 15 ignored by our optimization framework because of the lack of the IoT architecture and the framework, to meet their requirements. In Figure 10, we have compared the sensitivity of the layers used against the number of IoT requests processed and a number of IoT requests denied processing. As we can see from Figure 10, when three layers were used in an IoT network architecture, all the IoT requests were getting processed by our optimization framework, while the number of IoT requests denied processing was high when either of the fog or edge layer was not used in our IoT architecture. From Figure 11, we can conclude that the cost incurred in processing all the IoT requests was higher when all three layers, edge, fog, and cloud, were used. Hence we can conclude that all three layers must be utilized to make sure all the IoT requests are processed, but we would need to sacrifice cost incurred while doing so.

#### 5.2.4. Scenario 3: Allocation of IoT Requests on Basis of First Come First Serve (FCFS)

In this scenario, we have considered a three-layered IoT architecture, and no optimization framework was deployed, as shown in Figure 7, but all the nodes at all three layers were always active. Hence, when the 16 IoT jobs/requests described in Table 3 come in, they would be allocated to either node which was available, and no QoS metrics would be considered while allocating the IoT requests to the nodes at all the layers. As all 16 IoT requests were generated at the edge layer, of the 16 IoT requests received, only 13 IoT requests were executed, considering that each node was active and had infinite resource capacity and bandwidth to process each request. The IoT requests ignored were IoT request numbers 6, 8, and 16. In addition, the total cost incurred in processing the 16 IoT requests was 1230. However, if we consider that each node had limited and custom resource capacity and bandwidth that is listed in Figure 7 and all the nodes were active, then out of 16 IoT requests received by the IoT architecture, only 9 IoT requests were processed which are IoT request numbers 1, 2, 4, 5, 7, 11, 13, 15, 16 and the other 7 IoT requests were denied processing. Moreover, the total cost incurred in processing the IoT requests was 910. Hence we can conclude that if there was no usage of optimization framework, then the number of IoT requests denied processing would be high, and there would be no optimal usage of IoT architecture resources.

#### 5.2.5. Scenario 4: Allocation Based on Nearest Available Node

In this scenario, we consider a three-layered IoT architecture with no optimization framework deployed. We have considered the same data set as shown in Figure 7 wherein all the nodes were restricted by limited resources and connectivity. When an IoT job/request was received by our IoT framework, the request would be allocated to the node where the request was received, and if the node was incapable of processing the request, then it would be allocated to the nearest immediate adjacent node available. If the adjacent node was inactive, it will be activated immediately to process the request. Hence, when the 16 IoT jobs/requests described in Table 3 were received by our IoT framework, IoT request numbers 1, 2, 4, 6, 7, 8, 11, 13, 15, 16 were processed, and IoT request numbers 3, 5, 9, 10, 12, 14 were denied processing, as the nodes were not capable to process them. The total cost incurred by the framework was 830. Now, if we were to consider that all the nodes in the IoT architecture were always active and have unlimited resources to process each IoT request received. Hence, with the same incoming 16 IoT jobs/requests for the same data set described in Figure 7, the IoT architecture processed all the 16 IoT requests, and the total cost incurred was 1180.

**Figure 12:**
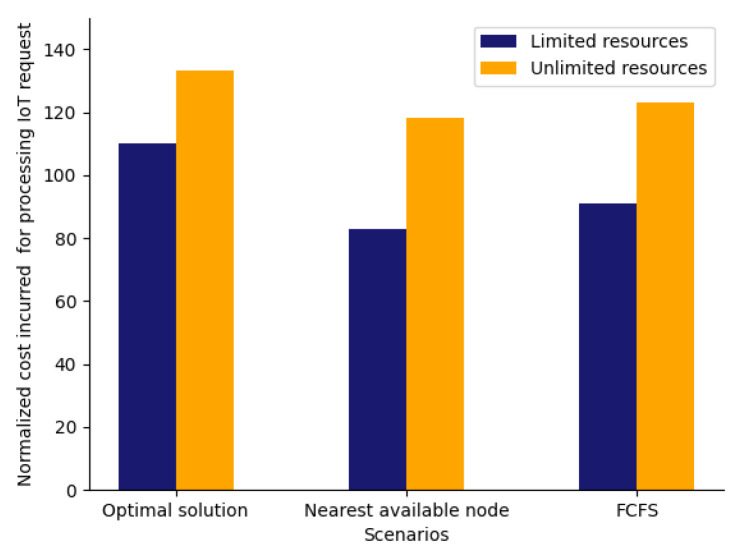
Cost comparison of Scenarios with unlimited and limited resources.

**Figure 13:**
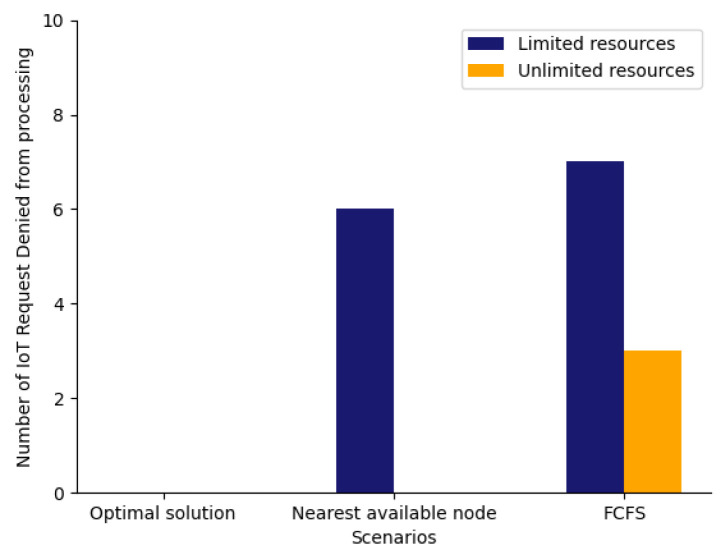
Scenario comparison with unlimited and limited resources against number of IoT request denied processing.

In Figure 12, we have compared the cost incurred for processing the IoT requests for scenarios like the optimal solution, nearest available node, and FCFS with limited and unlimited resources, respectively. We can see from Figure 12, that the cost incurred for the optimal solution with limited resources is average, and the least cost is incurred for the nearest available node. In addition for unlimited resources, the highest cost is incurred for the optimal solution. However, when we compare the number of IoT requests denied processing in Figure 13 for the same set of scenarios, it can be observed that it is zero for the optimal solution with both limited and unlimited resources while it is higher for other scenarios. Thus from Figure 12 and Figure 13 we can conclude that the best results are achieved for an optimal solution where the cost is average, and all the IoT requests are getting processed too.

Based on the assessment above, it can be inferred that the examined IoT network structure, in conjunction with the ILP framework, can efficiently distribute requests from diverse modern IoT use cases, like smart grids, e-Health, and autonomous vehicles. This is accomplished while minimizing energy costs and fulfilling both the scenarios constraints and communication limitations. Since integer linear programming cannot be solved in polynomial time, we utilized a CPLEX programming solver to determine solutions across diverse scenarios of an IoT network. The time taken to solve and produce a solution in CPLEX is heavily influenced by the dataset size and the computational ability of the machine executing the proposed framework. Consequently, in further research, a plan is to investigate heuristic approaches for optimal node selection in a three-layered IoT architecture and later evaluate the proposed framework using authentic live data obtained from a range of IoT applications and compare the outcomes.

## 6. Discussion

In this paper, we have examined the potential benefits of three layered IoT architectures with consideration of task offloading, node selection, and energy efficiency. Using a three-layered IoT architecture makes handling the increasing number of incoming IoT requests from upcoming advanced IoT use cases easier. With the implementation of task offloading and node selection mechanisms, processing IoT requests simultaneously is practically possible. Our proposed optimal node selection optimization framework ensures minimum energy is utilized while processing incoming IoT requests from advanced IoT use cases in addition to meeting the QoS metrics like resource capacity availability, latency, connectivity, bandwidth supported, servers availability and node activation.

Section 5 provided an elaboration on the significance of each layer with its cost relevance. Our framework gives us an estimate on time required to process the incoming IoT requests, but as the data-set size increases, the time required for processing all the IoT requests, will go up too. The limitation of our paper is the consideration of the mobility of the IoT request after being received at a node at the edge layer. Hence once an IoT request is received at an edge node at the edge layer, it is assumed that the IoT request stays steady at the exact location until it is processed.

## 7. Conclusions

This paper has explored the usage of a three layered IoT architecture including edge-fog-cloud, to promote modern advanced IoT use cases. We investigated how this generalized IoT architecture can be energy-efficiently used to process incoming IoT requests by optimally allocating the request to a node, at any given layer. We proposed an ILP-based optimal node selection framework to process all the incoming IoT requests energy efficiently while accommodating strict application-specific and network constraints. We have considered the energy costs for processing the IoT request at each layer and activating new servers and nodes at the fog and edge layers. For the implementation of our framework, we utilized CPLEX, and we assessed the practicality of our methodology by applying it to efficiently and optimally choose nodes for executing diverse IoT applications and their use cases, such as autonomous vehicles, smart grid, and eHealth, across various scenarios.

The results presented in this paper, highlight the importance of using all three layers in an IoT architecture along with the optimal node selection framework to achieve the required performance whilst minimising the energy usage. For example, the results showed that when using only two layers (cloud-fog or cloud-edge) in an IoT architecture with the optimal node selection framework, the majority of IoT requests were not being processed as the IoT architecture was unable to satisfy their requirements. We have also analyzed the impact of the energy cost at each layer on the optimal solution and on the performance of the IoT architecture. Overall, the results provided insight into the approaches and scenarios that can be used to achieve energy efficiency using a generic IoT architecture while serving diverse IoT use cases. Our future work will focus on using a heuristic approach to handle real-time node selection and measuring its performance against our proposed ILP framework.

## Figures and Tables

**Table 1:** Comparison of Energy optimization techniques in IoT architectures.

Ref.	Problem	Technique	Control Parameters	Outcome
[6]	Selecting radio frequency based visible light communication as an access point and meet the required QoS.	Markov determination procedure and replay of experience post determination and reinforcement learning methodology	Usage status of sub channel, quality, application types	Achieved energy efficiency with required data rate via network and sub channel selection
[27]	Allocation of energy efficient and delay restricted resource in fog	Deployment of application using Poly-time algorithm	Delay	Energy and time optimization
[30]	Fog computing being geographically distributed near end-users and restricted to sufficient services because of resource limitations	Linear programming	Low rental cost, minimum data	Resource optimization using collation of fog nodes and deployment of virtual machine
[4]	Deployment location for resource and application component in cloud-fog environment	Resource management layer using application placement and scheduling	Latency, network congestion, energy consumption and cost	Application placement optimization in IoT using edge and fog based architecture
[5]	Selection of suitable location for application module in fog-edge environment	ILP, analytical modelling, resource management framework	Delay, latency, energy usage	Module placement optimization in IoT using fog and edge based architecture
[29]	Ideal location in fog-cloud environment for application component	Module mapping algorithm	Delay, network usage, energy	Module placement optimization in IoT using fog and cloud based architecture
[28]	Suitable location in mobile-edge clouds for application or workload processing	Online approximation algorithms with polynomial-logarithmic (poly-log) competitive ratio for tree application graph placement	Latency, energy consumption, resource utilization	Workload placement optimization in IoT using edge cloud based architecture

**Table 2:** QoS metrics of IoT use-cases and its sub applications.

	Metric	Value
Smart Grid	Delay between devices	10 ms–1 ms
	Latency (end-to-end)	1 ms
	Teleprotection	≥10 ms
	Synchrophasor applications	≈20 ms
	SCADA and VoIP applications	100–200 ms
	Smart metering and others	upto few seconds
	Bandwidth/throughput	5–10 Mbps one control area and 25–75 Mbps for inter control
	Data rates/transmission rate	56 kbps–1 Mbps
	Reliability/availability	99–99.99%
Autonomous Vehicle	Delay between devices	≈1 ms
	Latency (end-to-end)	≈1 ms
	Bandwidth/throughput	512 Gbps–1024 Gbps
	Data rates/transmission rate	10–24 Gbps
	Reliability/availability	99.99–100%
e-Health	Delay between devices	1 ms–25 ms
	Latency (end-to-end)	1 ms–250 ms
	Bandwidth/throughput	5 Gbps–512 Gbps
	Data rates/transmission rate	5 Gbps–10 Gbps
	Reliability/availability	99.99–100%

**Table 3:** IoT Jobs considered for Scenario 4 and the optimal solution.

Job Number	Job Resource Requirement	Job Bandwidth	Job Latency	Job Origin	Optimally Selected Node
1	40	1000	10	12	12
2	30	1100	6	13	13
3	30	1100	65	13	20
4	80	60	8	11	5
5	60	70	100	16	6
6	40	1000	10	16	16
7	30	2000	10	17	17
8	60	80	8	17	4
9	35	2500	65	21	19
10	30	2000	65	23	15
11	25	1500	1	23	23
12	25	1500	110	18	13
13	25	750	6	20	21
14	45	900	60	14	11
15	60	70	100	10	7
16	50	85	100	11	3

## Data Availability

Not applicable.
